# LINKING MERIT-BASED INCENTIVE PAYMENT SYSTEM (MIPS) MEASURES TO AGE-FRIENDLY HEALTH SYSTEMS

**DOI:** 10.1093/geroni/igad104.1705

**Published:** 2023-12-21

**Authors:** Sara Murphy, Jennifer Severance, Sarah Ross, Kate Taylor, Janice Knebl

**Affiliations:** University of North Texas Health Science Center, Fort Worth, Texas, United States; University of North Texas Health Science Center, Fort Worth, Texas, United States; University of North Texas Health Science Center, Fort Worth, Texas, United States; University of North Texas Health Science Center, Fort Worth, Texas, United States; UNTHSC, Fort Worth, Texas, United States

## Abstract

IHI’s Age-Friendly Health Systems initiative provides a framework for older adult primary care focused on the 4Ms: Mentation, Mobility, Medications, and What Matters which addresses the need for an integrated system of care. Additionally, the Centers for Medicare & Medicaid Services have implemented merit-based incentivized payment system (MIPS) measures to promote provider practices that improve healthcare quality. MIPS measures and age-friendly care practices may be improved through training, which may lead to better patient outcomes. To integrate the 4Ms to improve care and patient safety, the Center for Older Adults at the University of North Texas Health Science Center standardize workflows and increase the number of annual wellness visits (AWV). A standardized template in the electronic medical record was created, questions in the patient registration software were added to identify eligible patients, and three different types of AWVs were standardized. Training courses were developed and completed by providers. The percentage of patients who receive an AWV is measured on an annual basis. Learnings have been integrated into training courses. Between July 2020 to June 2021, 297 healthcare professionals completed online training modules, with post-survey ratings showing helpful content with learning objectives being met. Provider support through training and incorporating MIPS measures and the Age-Friendly 4Ms framework into the AWV can support consistent outcome measurement and improve quality care for older adults.

